# [^68^Ga]Ga-PSMA-11 PET/CT and [^18^F]Fluorocholine PET/CT in Assessment and Clinical Decision Making of Recurrent Prostate Cancer: A Prospective Crossover Trial

**DOI:** 10.1007/s11307-025-02020-5

**Published:** 2025-05-28

**Authors:** Mohsen Beheshti, Malihe Shahbazi-Akbari, Marcus Hacker, Wolfgang Loidl, Werner Langsteger

**Affiliations:** 1 Division of Molecular Imaging and Theranostics, Department of Nuclear Medicne & Endocrinology, University Hospital Salzburg, Paracelsus Medical University, Salzburg, Austria; 2 Department of Nuclear Medicine & Endocrinology, PET/CT Center LINZ, Ordensklinikum, Linz, Austria; 3 Research Center for Nuclear Medicine, Tehran University of Medical Sciences, Tehran, Iran; 4 Division of Nuclear Medicine, Department of Biomedical Imaging and Image-Guided Therapy, Medical University of Vienna, Vienna, Austria; 5 Department of Urology, Ordensklinikum, Linz, Austria

**Keywords:** PET/CT, Prostate cancer, Biochemical recurrence, [^18^F]Fluorocholine, [^68^ Ga]Ga-PSMA-11

## Abstract

**Purpose:**

There are few prospective studies addressed toward the role of ^68^Gallium-labelled prostate-specific membrane antigen-11 ([^68^Ga]Ga-PSMA-11) compared to [^18^F]Fluorocholine ([^18^F]FCH) PET/CT in clinical decision-making as prostate-specific PET-tracers. This study aims to evaluate the impact of PET/CT using [^68^Ga]Ga-PSMA-11 and [^18^F]FCH in clinical management of recurrent prostate cancer (PCa) and correlates imaging findings with clinical characteristics of PCa.

**Procedures:**

Forty-six patients with PCa (mean age 68.3 ± 6.3 years) with biochemical recurrence were enrolled in this prospective crossover trial. All patients underwent both [^68^Ga]Ga-PSMA-11 and [^18^F]FCH PET/CT within a maximum interval of 12 days (median 7d). A standard randomization tool randomized the sequence of PET/CT imaging. Clinical decision-making occurred in an interdisciplinary meeting considering PET/CT findings. PET/CT-blinded readings were performed 3 months after imaging followed by a consensus meeting for final interpretation of detected lesions.

**Results:**

Both imaging modalities detected 136 total malignant lesions. [^68^Ga]Ga-PSMA-11 and [^18^F]FCH PET/CT detected 125 and 60 lesions with a sensitivity of 96% and 48%, respectively. Tumor-to-background ratios and semi-quantitative PET parameters on [^68^Ga]Ga-PSMA-11 were significantly higher in 54 (41.2%) tracer-avid congruent lesions detected on both imaging modalities. [^68^Ga]Ga-PSMA-11 PET/CT exclusively detected 71 (52.2%) lesions, while 6 (4.4%) lesions were solely seen on [^18^F]FCH PET/CT.

[^68^Ga]Ga-PSMA-11 and [^18^F]FCH PET/CT were positive in 35/46 (76%) and 26/46 (57%) patients, respectively. PET/CT imaging led to a major treatment change in 4 (8.7%) patients, of which [^18^F]FCH PET/CT had superior impact in one patient.

**Conclusions:**

[^68^Ga]Ga-PSMA-11 PET/CT revealed superior diagnostic performance to [^18^F]FCH PET/CT in patients with recurrent PCa, specifically with very low PSA levels ≤ 1 ng/ml. Moreover, it led to more accurate staging and clinical management of the disease. [^18^F]FCH PET/CT may play a complementary role in rare, select high-risk cases with negative [^68^Ga]Ga-PSMA-11 PET/CT and ongoing ADT.

**Supplementary Information:**

The online version contains supplementary material available at 10.1007/s11307-025-02020-5.

## Introduction

A total of 1,414,259 new cases of prostate cancer (PCa) and 375,304 related deaths were reported throughout the world in 2020 [1]. Management improvements resulted in stabilized or reduced incidence of PCa alongside decreased cancer-related mortality [2]. Hybrid imaging with radiolabeled prostate-specific membrane antigen (PSMA) and choline have been extensively used in assessment of patients with PCa in a wide variety of clinical scenarios. Using these imaging modalities with different kinetics for clinicians can enhance patient management by providing more confident reports.

Previous studies have shown that choline sensitivity in PCa depends upon tumor cell metabolic status and on size of the recurrent lesions [3]. [^18^F]/[^11^C]-Choline PET/CT sensitivity decreases at low PSA levels [4–6], and recommendations for using this modality are when PSA is > 1 ng/mL [7–9]. Tumor heterogeneity resulting from a variety of biological features such as PSMA expression, choline phosphorylation, and choline transport as well as prior treatments could affect PET tracer uptake. Early detection of recurrence in PCa, when the lesions are still sub-centimetric in biochemical recurrence (BCR), is crucial and a notable challenge from the perspective of diagnostic molecular imaging [10].

Studies on the role of PET/CT imaging with novel radiotracers like [^68^Ga]Ga-PSMA and [^18^F]Fluorocholine ([^18^F]FCH) have been most frequently done with a retrospective lens, and still there is a necessity for large prospective studies [3, 11].

To date, the superiority of [^68^Ga]Ga-PSMA as a PCa-specific agent for imaging and treatment over [^18^F]FCH is well known [10]. Based on multiple roles of choline in tumoral cell development [12], increased choline metabolism is not an absolute characteristic of PCa, especially in low-grade tumors. Diagnostic evidence shows [^18^F]/[^11^C]-choline PET/CT has higher sensitivity than [^68^ Ga]Ga-PSMA in the setting of restaging, compared to initial staging where [^68^Ga]Ga-PSMA has higher sensitivity. Nevertheless, meticulous patient selection is the most considerable issue to decrease false negative results and should be confined to high-risk patients, trigger PSA levels ≥ 1 ng/ml, PSA doubling time ≤ 6 months, and initial tumor stage ≥ pT3b or pN1 [9].

PSMA expression heterogeneity has been evaluated in metastatic lesions [13]. Overexpression of PSMA, even in poorly differentiated PCa is more frequent than choline metabolism; however, exclusively undifferentiated PCa or neuroendocrine phenotype of PCa could lose PSMA expression [3]. Moreover, oligometastatic disease is a favorable factor for planning local treatment in different types of cancer [14]. In metastatic PCa, attention to castration-sensitive oligometastatic versus castration-resistant oligoprogressive PCa is essential to alternate radical treatment (like stereotactic ablative radiation therapy) with systemic therapy (androgen deprivation therapy; ADT) to defer starting patients on ADT and avoiding ADT-associated complications [15].

Considering different tumor characteristics in PCa and various clinical situations of PCa patients with BCR [16] and the lack of ample prospective studies on [^68^Ga]Ga-PSMA compared to or in addition to [^18^F]FCH, we aimed to perform a cross-over randomized prospective study. This study evaluates the diagnostic performance of PET/CT scans using [^68^Ga]Ga-PSMA-11 and [^18^F]FCH within a short interval, assesses their impact on real-life clinical decision-making for recurrent PCa, and correlates imaging findings with the clinical characteristics of the disease and ongoing treatments.

## Materials and Methods

### Study Objectives

The primary objective of this study was to evaluate the diagnostic superiority of [^68^Ga]Ga-PSMA-11 over [^18^F]FCH PET/CT imaging in the detection of recurrence sites of prostate cancer after radical treatment. The secondary objective was to correlate the PET/CT organ specific imaging findings (i.e. local recurrence, lymph node metastases and visceral metastases) with clinical findings (i.e. trigger PSA and PSA doubling time) and ongoing ADT. In addition, the impact of [^68^Ga]Ga-PSMA-11 PET/CT over [^18^F]FCH PET/CT imaging on the therapeutic decision was assessed in this clinical trial. The details of primary and secondary objectives and endpoints are presented in Supplementary 1. The trial design is presented in Supplementary 2.

### Patients

The study was conducted in compliance with the protocol and in agreement with the Declaration of Helsinki, the International Conference on Harmonisation (ICH) Guidelines for Good Clinical Practice (GCP), and regulatory requirements as applicable. The study was also approved by the local ethics committee with the trial number EKS-10/18 and EudraCT number: 2017–005078-20. Signature of the written informed consent was obtained from all individuals.

This prospective, single-center, open-label, randomized, 2-armed crossover study was conducted in 46 patients with PCa presenting with biochemical recurrence (BCR). In each arm, 23 patients were included using a standard randomization tool for determining the sequence of PET/CT imaging. This randomized crossover design was used to ensure that no bias was introduced by the order of the two PET/CT scans and to minimize the confounding covariates and intra-patient variabilities. PET/CT imaging were performed in all patients applying both tracers in a maximum interval of 12 days (range: 5–12 days, median: 7 days). The patients were included in this trial between August 2019 and December 2021. Inclusion and exclusion criteria are noted in Supplementary 3. PSA at the day of PET/CT imaging was considered as trigger PSA (tPSA).

### Radiopharmaceuticals

#### [^68^Ga]Ga-PSMA-11

[^68^Ga]Ga-PSMA-11 was prepared using PSMA-11 Sterile Cold Kit (trade name: Illuccix®, Telix Pharmaceuticals Limited, Melbourne, Australia) and gallium-68 obtained from a ^68^Ge/^68^Ga generator (Galli Ad®, IRE ELIT, Fleurus, Belgium) under Good Manufacturing Practices (GMP) conditions in an in-house radiopharmacy. A [^68^Ga]Ga-PSMA-11 activity of 2.0 ± 10% MBq per kilogram (kg) of body weight (bw) was administered by intravenous injection as a single bolus.

#### [^18^F]Fluorocholine

[^18^F]-Fluoromethylcholine ([^18^F]FCH) (IASOcholine®, Curium Austria, Graz, Austria) was produced and supplied commercially. A [^18^F]FCH dosage of 4.0 ± 10% MBq/kg/bw was administered intravenously as a single bolus.

#### PET/CT Imaging

[^68^Ga]Ga-PSMA-11 and [^18^F]FCH PET/CT were performed within a maximum interval of 12 days (median 7 d). All study images were performed using a dedicated PET/CT scanner (Discovery 710; GE Healthcare, Milwaukee, WI, USA) with an extended field-of-view full-ring high-resolution Lutetium yttrium oxyorthosilicate (LYSO) PET component and a 128-slice spiral CT. Image acquisition was done for all patients 60 min (min.) after intravenous (i.v.) injection of the radiotracer. As a routine protocol in our department, all patients received a standard volume (250 mL) of sodium chloride i.v. infusion and 250–500 mL of oral fluid. Standard whole-body acquisitions were obtained from the base of the skull to the proximal thigh with an acquisition time per bed position of 2.5 min. using time of flight mode. Images were reconstructed identically using the ordered-subsets expectation maximization algorithm (4 iterations, 18 subsets), then subsequently using a post-reconstruction smoothing gaussian filter (4.0 mm full-width at half-maximum). A contrast-enhanced-CT (CE-CT) scan with a high beam tube-current modulation (120–330 mA, 0.6 s per rotation, 5.0 mm reconstructed section thickness, 0.5 mm overlap, 512_512 matrix, pitch index 1.5) was completed in all patients, either in [^18^F]FCH or in [^68^Ga]Ga-PSMA-11 PET/CT. Low beam current modulation (80–120 mA) was used for the CT portion of the second PET/CT. Reformatted transverse, coronal and sagittal views were analyzed for interpretation. Advanced PET/CT software (AW-4.6; GE Medical Systems, Milwaukee, WI, USA) that allows PET, CT and fusion PET/CT data to be viewed simultaneously was used for reading. No major protocol deviations have been reported.

Semi-quantitative PET measurements were performed by drawing a fixed sized volume of interest (VOI) of 25 mm diameter over suspicious lesions. Furthermore, background tracer uptake in soft tissue was evaluated by drawing a standard reference VOI on the right gluteus muscle, sparing intramuscular vessels.

#### Final Interpretation

PET/CT readings were performed 3 months after imaging by two experienced nuclear medicine specialists (MB & WL), who were only aware of clinical diagnosis. In case of discrepancies between two readers, images were reviewed again together with a third independent expert (MH). The final diagnosis of each detected lesion was defined in a consensus multidisciplinary meeting. Each lesion detected on a single PET/CT scan was compared with corresponding images using another tracer and classified as a congruent or incongruent lesion.

The assessment of lesions was based a “standard of truth” to ascertain the positivity of lesions detected. The standard of truth was based on the lesion type as follows:

- *Local recurrences, lymph-node- and visceral-metastases:* the final diagnosis of a local recurrence and/or positive lymph node on [^68^Ga]Ga-PSMA-11 and [^18^F]FCH PET studies was principally based on histopathological findings. However, in the majority of cases with no histopathology, lesions were considered malignant if they were positive on both [^68^Ga]Ga-PSMA-11 and [^18^F]FCH PET with corresponding pathological findings on CT. Otherwise, detected lesions had to be confirmed at a follow-up examination. Lesions with increased tracer uptake were considered malignant if they showed persistent or increased uptake on follow-up PET scans consistent with morphological changes on CT or if decreased tracer activity was seen on treatment correlating with clinically regressive findings.
- *Bone metastases:* Lesions were considered bone metastases if they were positive both on [^68^Ga]Ga-PSMA-11 and [^18^F]FCH PET and CT with corresponding positive uptake in an additional bone-seeking modality (i.e., [^18^F]-Fluoride PET/CT or bone scan) or positive MRI. In case of [^68^Ga]Ga-PSMA-11 PET and CT discrepancies, further verification on clinical and imaging follow-up examinations after 3 months at the earliest (range, 3–6 months) was required. Persisting lesions with increased uptake on functional imaging modalities (i.e., [^18^F]-Fluoride, [^68^Ga]Ga-PSMA-11 or [^18^F]FCH PET) and/or corresponding malignant morphological changes on anatomical imaging (MRI or CT) with clinical evidence of disease progression were considered malignant.

#### Safety Evaluation

Safety evaluation (analysis of the frequency, severity and relationship to study interventions of adverse events (AEs) and serious AEs (SAEs) throughout the patient’s participation in the study) was performed for all patients after administration of [^68^Ga]Ga-PSMA-11 and [^18^F]FCH, at 24 h post-administration, Day 30, and Day 60.

## Statistical Analysis

The study initially estimated a sample size of 38 patients based on literature assumptions to compare lesion detection between two radiotracers [10, 26]. The calculation assumed an average of 10 lesions per patient with [^18^F]FCH and 13 with [^68^Ga]Ga-PSMA-11 PET/CT, with a standard deviation of 5. An assumed 20% dropout rate led to a final enrollment target of 46 patients. The robustness of the sample size was validated through simulations using a bivariate negative binomial probability function, with estimates drawn from previous studies. The primary endpoint was analyzed with a likelihood-based estimation and inference, using the “missing at random” assumption. The variables ‘imaging method,’ ‘period,’ and ‘patient’ were used as explanatory variables in the model. The difference in the number of PCa lesions between the two imaging methods estimated by the model was displayed with a 95% confidence interval and a p-value. Sensitivity, specificity, PPV and NPV calculations did not take within-patient or within-regional correlations into account. Data was analyzed with SAS Version 9.4.

## Results

### Study Cohort

Our study consisted of 46 PCa patients (mean age: 68.3 ± 6.3) with BCR who underwent both [^68^Ga]Ga-PSMA-11 and [^18^F]FCH PET/CT. Patient characteristics are summarized in Supplementary 4. tPSA level ranged from 0.2 to 9 ng/ml (Supplementary 5) with a median of 0.7 ng/ml. Details of tPSA, PSA doubling time (PSAdt) and PSA velocity in patients with metastases are demonstrated in Supplementary 5. In our study, 27 patients had radical former treatment (RPE ± RT; non-ADT group), and 19 patients had radical plus systemic (RPE ± RTx + ADTongoing ± CTx) former treatment (ADT group). Patients in the ADT group received ADT (> 6 months = long-term) prior to the study and did not discontinue ADT treatment during PET/CT scans.

### Primary Objective

The diagnostic superiority of [^68^Ga]Ga-PSMA-11 over [^18^F]FCH PET/CT imaging in the detection of prostate cancer recurrence was assessed at patient- and lesion-level.

In the patient-based analyses, [^68^Ga]Ga-PSMA-11 and [^18^F]FCH PET/CT had overall detection rates of 76% (35/46) and 57% (26/46), respectively (Tables 1 and 2).

**Table 1:** Detection rate of [^68^Ga]Ga-PSMA-11 and [^18^F]FCH PET/CT scans

Detection rate	Median detected lesion per patient	Overall	tPSA ≤ 1ng/ml	1 < tPSA ≤ 2ng/ml	tPSA > 2ng/ml	Non-ADT group	ADT group
[^18^F]FCH PET/CT	1 (range 0–6)	26/46 (57%)	9/27 (33%)	5/6 (83%)	12/13 (92%)	10/27 (37%)	16/19 (84%)
[^68^Ga]Ga-PSMA-11 PET/CT	2 (range 0–10)	35/46 (76%)	16/27 (59%)	6/6 (100%)	12/13 (92%)	18/27 (67%)	17/19 (89%)

**Table 2:** Detection rate of [68Ga]Ga-PSMA-11 and [18F]FCH PET/CT scans in different PSA levels

Trigger PSA	PSMA positive	PSMA Detection rate	FCH positive	FCH Detection rate	Total
PSA ≤ 0.5	7	7/12 (58%)	4	4/12 (33%)	12
0.5 < PSA < 1	9	9/14 (64%)	5	5/14 (36%)	14
1 ≤ PSA < 2	5	5/5 (100%)	4	4/5 (80%)	5
PSA ≥ 2	14	14/15 (93%)	13	13/15 (87%)	15
Total	35	35/46 (76%)	26	26/46 (57%)	46

In the lesion-based analyses, 136 lesions were ultimately classified as malignant based on final consensus expert review, including 130 and 65 lesions in [^68^Ga]Ga-PSMA-11 and [^18^F]FCH PET/CT scans, respectively. There were 5 densely sclerotic bone lesions on 1 patient’s CT images without tracer uptake in both PET modalities. The number and classification of lesions are given in Table 3.

**Table 3:** Details of lesions classification and anatomic location of metastases

Location of lesions	[^18^F]FCH + ve lesions	[^68^Ga]Ga-PSMA-11 + ve	Total lesions	incongruent [^18^F]FCH + ve	incongruent [^68^Ga]Ga-PSMA-11 + ve	Total incongruent lesions	Total congruent lesions
**Total**	**60**	**125**	**131**	**6/77 (7.8%)**	**71/77 (92.2%)**	**77 (100%)**	**54 (100%)**
Local Recurrence	2	4	4	0 **(0%)**	2 **(2.6%)**	2 **(2.59%)**	2 **(3.7%)**
Regional Lymph Node	21	36	39	3 **(5.1%)**	18 **(%)**	21 **(27.3%)**	18 **(33.3%)**
Distant Metastasis (M1)	37	85	88	3 **(5.1%)**	51 **(%)**	54 **(70.1%)**	34 **(63%)**
Bone-Distant Metastasis	10	20	21	1 **(1.3%)**	11 **(14.2%)**	12 **(15.6%)**	9 **(16.7%)**
Non-bone-Distant Metastasis	27	65	67	2 **(2.6%)**	40 **(51.9%)**	42 **(54.5%)**	25 **(46.3%)**
Distant Lymph Node Metastasis	17	53	55	2 **(2.6%)**	38 **(49.4%)**	40 **(51.9%)**	15 **(27.8%)**
Distant Lung Metastasis	10	12	12	0 **(0%)**	2 **(2.6%)**	2 **(2.6%)**	10 **(18.5%)**

*(*+ *ve* = *positive)*

**Fig. 1.**
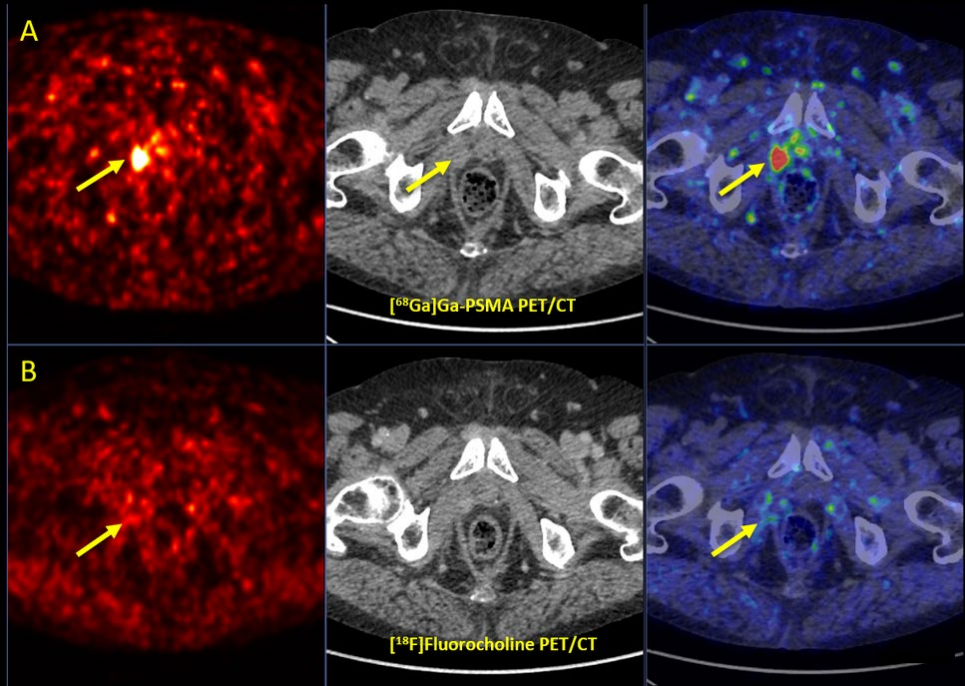
**A** [^68^Ga]Ga-PSMA and **B** [^18^F]Fluorocholine PET/CT in a prostate cancer patient with biochemical recurrence (tPSA: 1.53 ng/ml, PSAdt: 11 months) after RPE, RTx and ADT. [^68^Ga]Ga-PSMA (upper row) PET/CT (transaxial PET (left), CT (middle), and fusion PET/CT (right) images) shows focal uptake (SUVmax = 24.0) in the right prostatic bed (arrows), suggesting local recurrence. No [^18^F]Fluorocholine (lower row) uptake. *RPE* Radical Prostatectomy; *RTx* Radiotherapy; *ADT* Androgen Deprivation Therapy; *tPSA* Trigger Prostate Specific Antigen; *SUV max* Maximum Standardized Uptake Value; *PSMA* Prostate-Specific Membrane Antigen, *PET/CT* Positron Emission Tomography/Computed Tomography

**Fig. 2.**
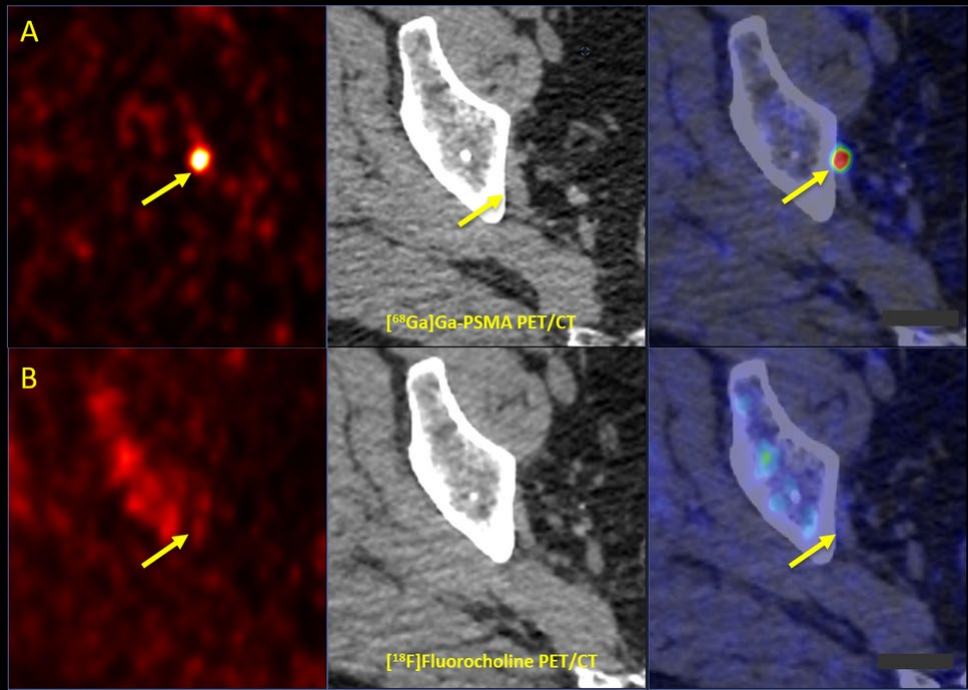
**A** [^68^Ga]Ga-PSMA and **B** [^18^F]Fluorocholine PET/CT in a prostate cancer patient with biochemical recurrence (Gleason score 3 + 4, tPSA: 1.85 ng/ml, PSAdt: 12 months) after RPE, RTx and ADT. [^68^Ga]Ga-PSMA (upper row) PET/CT (transaxial PET (left), CT (middle), and fusion PET/CT (right) images) shows focal uptake (SUV max = 16.2) in a right external iliac lymph node (diameter: 6 mm, arrows) suggesting lymph node metastasis. No [^18^F]Fluorocholine (lower row) uptake. *RPE* Radical Prostatectomy, *RTx* Raditherapy, *ADT* Androgen Deprivation Therapy, *tPSA* Trigger Prostate Specific Antigen, *SUV max* Maximum Standardized Uptake Value, *PSMA* Prostate-Specific Membrane Antigen, *PET/CT* Positron Emission Tomography/Computed Tomography

[^68^Ga]Ga-PSMA-11 PET/CT was able to detect significantly higher number of lesions compared to [^18^F]FCH PET/CT (125 vs. 60, respectively; P-value < 0.001) with sensitivity 0.96 (95%CI: 0.91; 0.98) and AUC 0.98 (95%CI: 0.96; 1.00) in [^68^Ga]Ga-PSMA-11 PET/CT (P-value < 0.001), and sensitivity 0.48 (95%CI: 0.40; 0.56) and AUC 0.74 (95%CI: 0.70; 0.78) in [^18^F]FCH PET/CT (P-value < 0.001) (Figs. 1 & 2).

### Secondary Objectives

#### Correlation with Clinical Findings and Ongoing Treatment

In 10/46 (22%) patients with negative PET imaging using both radiotracers, tPSA was ≤ 0.77 ng/ml (range: 0.26–2.92 ng/mL, median: 0.54 ng/mL), and 9 (90%) of these patients were in the non-ADT group. In 10/46 (22%) patients with only positive [^68^Ga]Ga-PSMA-11 PET/CT, 8 patients had tPSA ≤ 0.89 ng/mL (range: 0.17–5.38 ng/mL, median: 0.49 ng/mL) and were in the non-ADT group.

Among 35 patients with reported PSAdt, 15 (43%) had PSAdt ≤ 6 months, of whom 11 (73%) showed positive PET/CT with both tracers (Supplementary 5). In 26 [^18^F]FCH-positive patients ([^68^Ga]Ga-PSMA-11 PET/CT was negative in only one), most had features of advanced disease (GS ≥ 7, ISUP ≥ 4, higher initial pathologic staging, on ADT) and higher tPSA levels (median 1.7 ng/ml).

Overall, 54 congruent and 77 incongruent lesions were found between two imaging modalities (71 incongruent [^68^Ga]Ga-PSMA-11-avid lesions and 6 incongruent [^18^F]FCH-avid lesions). Interestingly, all incongruent [^18^F]FCH positive lesions were seen in the ADT group. However, all incongruent [^68^Ga]Ga-PSMA-11-avid lesions were observed in patients in the non-ADT group.

#### Comparison of Tracer Intensity

**Fig. 3.**
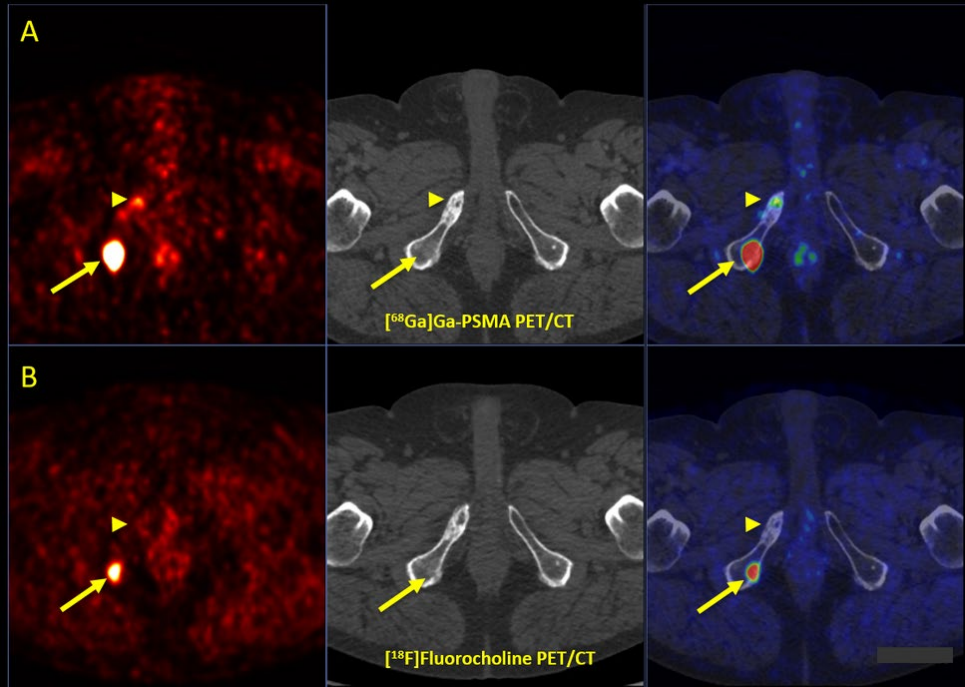
**A** [^68^Ga]Ga-PSMA and **B** [^18^F]Fluorocholine PET/CT in a prostate cancer patient with biochemical recurrence (Gleason score 4 + 4, tPSA: 3.95 ng/ml, PSAdt: 1 months) after RPE, CTx and ADT. Superior impact of [^68^Ga]Ga-PSMA PET/CT compared to [^18^F]Fluorocholine PET/CT in bone metastasis. (**A**) [^68^Ga]Ga-PSMA (upper row) PET/CT (transaxial PET (left), CT (middle), and fusion PET/CT (right) images) represents focal uptake (SUV max = 65.4) in the right ischium (arrows) with extremely low [^18^F]Fluorocholine (lower row) uptake (SUV max = 16.8). In addition, [^68^Ga]Ga-PSMA (upper row) PET/CT depicts focal uptake (SUV max = 6.7) in the right ischium (arrowheads) with no [^18^F]Fluorocholine (lower row) uptake. *RPE* Radical Prostatectomy, *CTx* Chemotherapy, *ADT* Androgen Deprivation Therapy, *tPSA* Trigger Prostate Specific Antigen, *SUV max* Maximum Standardized Uptake Value, *PSMA* Prostate-Specific Membrane Antigen, *PET/CT* Positron Emission Tomography/Computed Tomography

The average lesion SUV_max_ in [^68^Ga]Ga-PSMA-11 PET/CT imaging was approximately two-fold that of [^18^F]FCH PET/CT (12.81 ± 13.5 vs. 6.35 ± 4.89) (Fig. 3). The average tumor-to-background ratio (TBR) in [^68^Ga]Ga-PSMA-11 PET/CT scans was approximately three-fold that of [^18^F]FCH PET/CT scans (9.16 ± 10.93, vs. 2.55 ± 2.59).

#### Molecular and Structural Patterns of Lesions

**Fig. 4:**
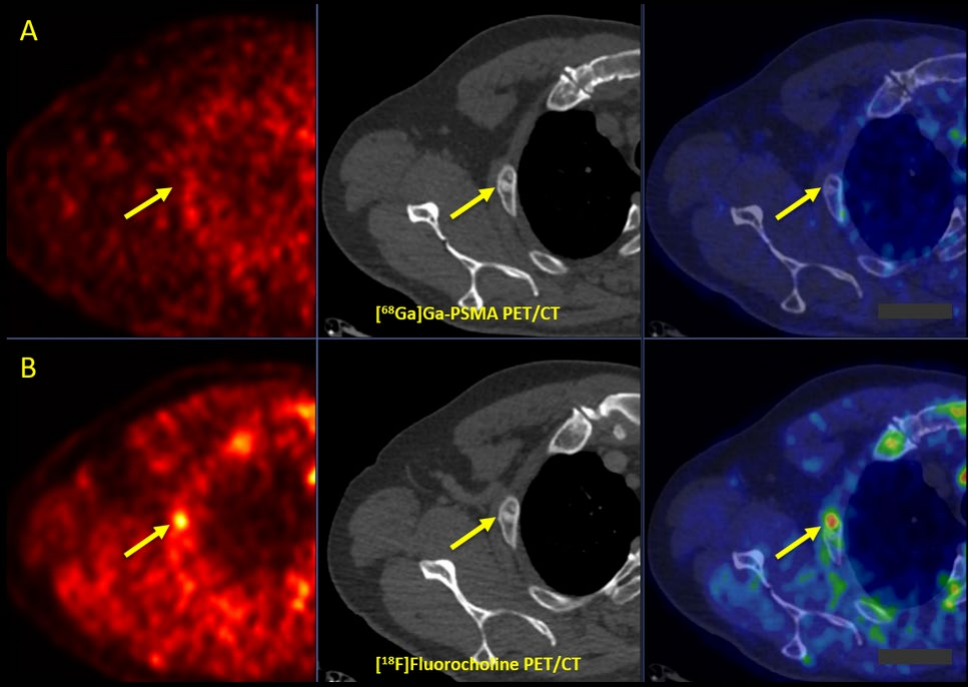
**A** [^68^Ga]Ga-PSMA and **B** [^18^F]Fluorocholine PET/CT in a prostate cancer patient with biochemical recurrence (Gleason score 4 + 4, tPSA: 4.90 ng/ml, PSAdt: 12 months) after RTx and ADT. Superior impact of [^18^F]Fluorocholine PET/CT (lower row, arrows) compared to [^68^Ga]Ga-PSMA PET/CT (upper row) in the assessment of a bone metastasis in the 2nd right rib. *RPE* Radical Prostatectomy, *CTx* Chemotherapy, *ADT* Androgen Deprivation Therapy, *tPSA* Trigger Prostate Specific Antigen, *SUV max* Maximum Standardized Uptake Value, *PSMA* Prostate-Specific Membrane Antigen, *PET/CT* Positron Emission Tomography/Computed Tomography

Among 12 incongruent bone metastases, one bone lesion was only [^18^F]FCH positive (Fig. 4), and 11 bone lesions were exclusively [^68^Ga]Ga-PSMA-11 positive (Table 3). Notably, 8/12 (67%) of bone metastases were bone marrow metastases, revealing no underlying findings on CT, which were not detectable by [^18^F]FCH PET/CT. We found that 78% (56/71) of incongruent [^68^Ga]Ga-PSMA-11 avid lesions were ≤ 1 cm on CT images. However, [^18^F]FCH-PET was able to detect only 3 (3/6, 50%) sub-centimetric incongruent bony lesions.

#### TNM Restaging, PET/CT Influence on Treatment Approach

Concordant TNM restaging was seen in 29 patients (63%). The treatment approach consisted of minor changes in 2 (4.3%) and major changes in 4 (8.7%) patients. [^18^F]FCH PET/CT and [^68^Ga]Ga-PSMA-11 PET/CT scans revealed superiority for defining treatment approach in one and three patients, respectively.

From 27 (27/46, 59%) patients with distant metastases based on recent PET/CT restaging, 52% (14/27) demonstrated oligometastases (i.e. ≤ 3 lesions, low-volume based on CHAARTED criteria) [17, 18]. Furthermore, the treatment plan was exclusively changed from radical alone to starting ADT in one low-volume oligometastatic patient and one high-volume polymetastatic patient. No notable difference was observed between [^18^F]FCH and [^68^Ga]Ga-PSMA-11 PET/CT in terms of their impact on major treatment changes.

#### Safety

There were no serious or fatal adverse events in any patient due to either tracer injection. Exclusively, we found one patient with an adverse event (pruritus in extremities) after the administration of [^68^Ga]Ga-PSMA-11 injection with no evidence of complication during patient follow-up. There was no study discontinuation due to an adverse event.

## Discussion

We report positive results from a prospective trial with novel correlation of PET-imaging findings with ongoing ADT, clinical parameters, and morphological changes on CT to assess associations between specific disease characteristics and PET positivity by radiotracer. Our findings add to the current literature, with a majority of reported results comparing the diagnostic performance of [^68^Ga]Ga-PSMA-11 and [^18^F]FCH PET/CT from retrospective studies [3–5, 10, 11, 14, 19–24] with limited data from prospective studies [25].

Our results show that [^68^Ga]Ga-PSMA-11 has higher diagnostic performance compared to [^18^F]FCH in PCa patients, even when PSA levels are low (i.e., ≤ 1 ng/ml). In line with previous investigations, our results demonstrate superior diagnostic performance of [^68^Ga]Ga-PSMA-11 compared to [^18^F]FCH PET/CT, particularly in patients with low tPSA values (i.e. ≤ 1) [3–5, 10, 11, 14, 19–24, 26]. Overall, [^68^Ga]Ga-PSMA-11 PET/CT demonstrated higher detection of local and distant metastatic disease. PCa lesions presented with higher contrast and uptake with [^68^Ga]Ga-PSMA-11 than with [^18^F]FCH PET/CT, which contributed to the higher detection rate of [^68^Ga]Ga-PSMA-11 PET/CT at low PSA serum values. Moreover, it led to more accurate staging and clinical management of disease and has an acceptable safety profile. Results correspond with recent consensus documents that recommend radiolabeled PSMA as the preferred PET radiotracer in PCa patients [27].

The effects of ongoing or prior treatments on [^18^F]FCH and [^68^Ga]Ga-PSMA-11 PET/CT diagnostic performance has not been fully elucidated [28–31]. Some previous studies report no effect of ADT on [^18^F]FCH uptake in hormone-refractory PCa cases [28, 29]. In contrast, other studies demonstrate ADT suppression of choline uptake in castration-sensitive PCa patients in preference to castration-resistant disease [32] [33] [34]. Luca Urso et al. reported that [^18^F]FCH PET/CT assists to identify metastatic PCa in previously castration-sensitive disease that has switched to castration-resistant. In our investigation, 16/19 (84%) patients on ADT had positive [^18^F]FCH PET/CT, and both imaging modalities showed high and comparable detection rate (> 80%) in patients with ongoing ADT (ADT group). However, higher levels of tPSA were seen in the ADT group contrary to the non-ADT group, which relies on greater predictive impact of tPSA on PET/CT positivity. Moreover, all incongruent [^18^F]FCH-avid lesions were found in the ADT group. In the non-ADT group, all incongruent lesions were only [^68^Ga]Ga-PSMA-11 avid. These findings support the idea that ADT does not limit [^18^F]FCH-avid lesion detection, and [^18^F]FCH could have added value in patients with higher levels of tPSA who are receiving ADT.

Our results demonstrating statistically significant (P-value < 0.001) superiority of [^68^Ga]Ga-PSMA for greater lesion detection (about twofold of [^18^F]FCH-avid lesions) is consistent with previous studies [10, 11, 26, 35]. In addition, [^68^Ga]Ga-PSMA-11 had higher detection than [^18^F]FCH PET/CT for bone marrow metastases (8 vs 0 lesions, respectively) and small sub-centimetric lesions (84 vs 31 lesions, respectively), particularly in patients with low tPSA (≤ 1.0 ng/ml). These results are in line with findings of previous studies related to higher uptake of malignant lesions and lower background activity with [^68^Ga]Ga-PSMA-11 leading to better contrast [10, 11, 14, 26].

Previous studies reported that [^18^F]/[^11^C]-Choline PET revealed better performance in re-staging of selected high-risk patients with tPSA levels ≥ 1 ng/ml, PSA doubling time ≤ 6 months, and initial tumor stage ≥ pT3b or pN1 [3, 9, 36]. In a sub-group of our patients with PSAdt ≤ 6 months PET/CT using both tracers were positive in 11/15 (79%) patients even in 9/15 (60%) patients with tPSA < 1 ng/ml. These findings may rely on the complementary role of PSAdt on decision making for diagnostic imaging in individual cases with low tPSA and ongoing ADT. These findings are in line with recent data on [^68^Ga]Ga-PSMA-11 PET/CT-guided external beam radiation therapy (EBRT), which appeared to improve BCR-free survival compared to [^18^F]FCH PET/CT-guided EBRT. Similarly, PSAdt at recurrence appeared to be a prognostic factor associated with treatment response [37].

In a systematic review, the authors highlighted beneficial impact of [^68^Ga]Ga-PSMA-11 PET to predict survival outcomes, particularly in oligometastatic patients [38]. We report ability of [^68^Ga]Ga-PSMA-11 PET/CT to identify seven more patients with oligometastatic disease and low total lesion volume (TLV), compared to [^18^F]FCH PET/CT, consequently leading to better detection of TLV and presumably more precise patient management with [^68^Ga]Ga-PSMA-11 PET/CT.

Although concordant TNM restaging was seen in 29/46 (63%) patients, [^18^F]FCH PET/CT showed limited performance in M staging of 10/17 (59%) patients. This may be related to low tPSA, early-stage disease (being castration-sensitive), high PSMA expression even in poorly differentiated PCa, tumor heterogeneity, sub-centimetric lesions and early bone marrow involvement.

A recently published study on the superior impact of [^18^F]PSMA-1007 PET/CT on diagnostic thinking (impact of a test result on posttest vs. pretest probability of a correct diagnosis) and therapeutic decision making (description and quantification of impact of diagnostic information gained with both radiopharmaceuticals on patient management) compared to [^18^F]FCH PET/CT [25]. An earlier study comparing [^68^Ga]Ga-PSMA-11 to [^18^F]fluoromethylcholine in patients with BCR and low PSA found a major or moderate impact on management in 24 cases (63%), and 54% (13/24) of those impacts were attributable to the findings on [^68^Ga]Ga-PSMA-11 imaging alone [26]. In addition, several studies have shown [^68^Ga]Ga-PSMA-11 has an impact on clinical management, with one study finding 75% of BCR cases had a change in management [39, 40]. Our results showed superiority for defining treatment approach in one patient with [^18^F]FCH PET/CT and three patients with [^68^Ga]Ga-PSMA-11 PET/CT, but no significant difference between [^18^F]FCH and [^68^Ga]Ga-PSMA-11 PET/CT in change in treatment approach for patients with oligometastatic disease. This may be related to the higher number of included patients and follow-up strategies of the mentioned studies.

The prospective study design lends to the strength of our study; however, limitations include limited histopathological verification of lesions and missing long-time follow-up data for some patients.

## Conclusion

We report results from a prospective study demonstrating the superior diagnostic performance of [^68^Ga]Ga-PSMA-11 PET/CT over [^18^F]FCH PET/CT in patients with BCR of PCa, specifically in cases with very low PSA levels (i.e. ≤ 1 ng/ml). Moreover, [^68^Ga]Ga-PSMA-11 PET/CT led to more accurate staging and clinical management of the disease. [^18^F]FCH PET/CT may play a complementary role only in rare, selected high-risk cases with negative [^68^Ga]Ga-PSMA-11 PET/CT with ongoing ADT.

## Supplementary Information

Below is the link to the electronic supplementary material.

Supplementary file1 (DOCX 16 KB)

Supplementary file2 (DOCX 368 KB)

Supplementary file3 (DOCX 20 KB)

Supplementary file4 (DOCX 15 KB)

Supplementary file5 (DOCX 20 KB)

## Data Availability

The datasets generated and/or analyzed during the current study are available from the corresponding author on reasonable request.
