# Supplementary material for: [68Ga]Ga-PSMA-11 PET/CT and [18F]Fluorocholine PET/CT in Assessment and Clinical Decision Making of Recurrent Prostate Cancer: A Prospective Crossover Trial
Source: Mol Imaging Biol. 2025 May 28;27(4):597–605. doi: 10.1007/s11307-025-02020-5 (PMC12405339; doi:10.1007/s11307-025-02020-5)
Supplement: Supplementary file 1 — Supplementary file1 (DOCX 16 KB) [file 11307_2025_2020_MOESM1_ESM.docx]

**Supplementary 1:**

Primary and secondary objectives and endpoints of the study

| Primary Objective | To evaluate the diagnostic superiority of [^68^Ga]Ga-PSMA-11 over [^18^F]FCH PET/CT imaging in the detection of recurrence sites of prostate cancer after radical treatment. |
| --- | --- |
| Secondary Objectives | - To compare the diagnostic performance of [^68^Ga]Ga-PSMA-11 and [^18^F]FCH PET/CT imaging. - To correlate the PET/CT organ specific imaging findings (i.e. local recurrence, lymph node metastases and visceral metastases) with clinical findings (i.e. trigger PSA and PSA doubling time) and ongoing androgen deprivation treatments. - To compare the impact of [^68^Ga]Ga-PSMA-11 PET/CT over [^18^F]FCH PET/CT imaging on the therapeutic decision (minor and/or major therapeutic changes with regards to the number and/or lesion(s) location(s)) - Safety evaluation |
| Primary endpoint | Comparison of the number of correctly identified prostate cancer lesions, as confirmed by a Consensus/Expert meeting, detected using [^68^Ga]Ga-PSMA-11 PET/CT and [^18^F]FCH PET/CT imaging. |
| Secondary endpoints | - SUVmax and tumor-to-background ratios of the metastatic lesions in each organ as assessed by [^68^Ga]Ga-PSMA-11 and [^18^F]FCH PET/CT. - Determination of the number of patients with changes in TNM-restaging and/or treatment following the results of the different PET/CT imaging scans assessment. - Number of patients with incongruent findings on [^68^Ga]Ga-PSMA-11 PET/CT and [^18^F]FCH PET/CT - Determination of the number of detection lesions in patients with low PSA (i.e. <1.0 ng/ml) on each PET/CT modality. - Determination of number of detected lesions in patients with ongoing and without androgen deprivation treatment (ADT) - Sensitivity, specificity and AUC values for each PET/CT imaging tracer as determined on three levels: lesion and patient level. - Number of adverse events or serious adverse events in relation with the study drug (description of the adverse events, incidence, severity and causality). |
