# Supplementary figures and images for: [68Ga]Ga-PSMA-11 PET/CT and [18F]Fluorocholine PET/CT in Assessment and Clinical Decision Making of Recurrent Prostate Cancer: A Prospective Crossover Trial
Source: Mol Imaging Biol. 2025 May 28;27(4):597–605. doi: 10.1007/s11307-025-02020-5 (PMC12405339; doi:10.1007/s11307-025-02020-5)

**Supplementary 2:**

Study design flowchart.


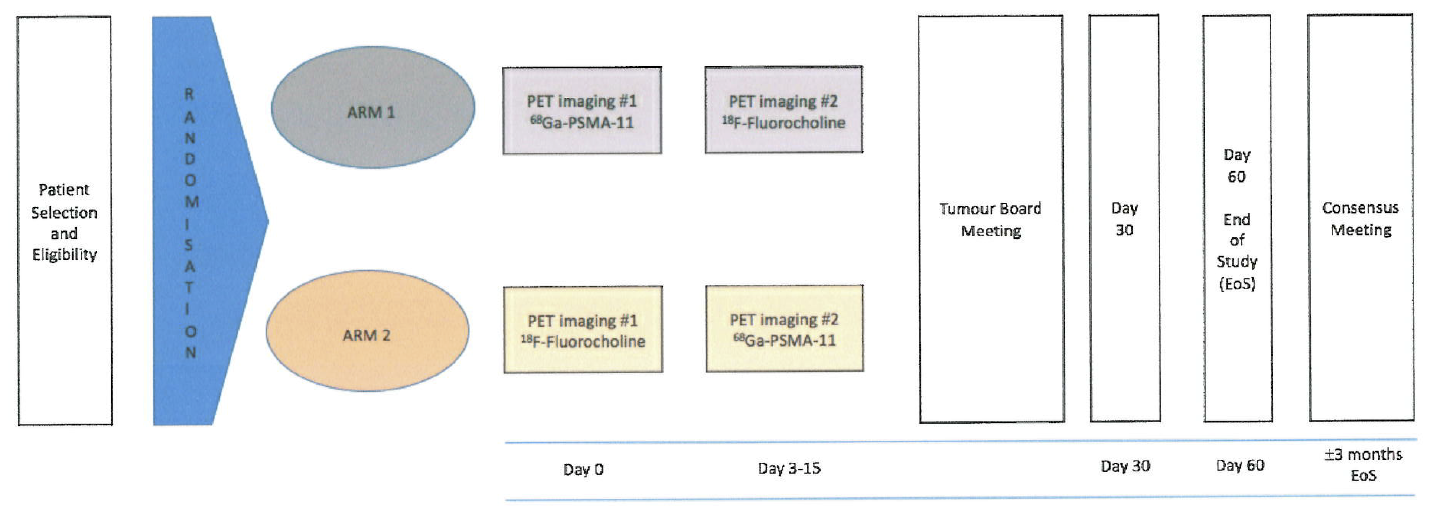

Supplement: Supplementary file 2 — Supplementary file2 (DOCX 368 KB) [file 11307_2025_2020_MOESM2_ESM.docx]
