# Supplementary material for: [68Ga]Ga-PSMA-11 PET/CT and [18F]Fluorocholine PET/CT in Assessment and Clinical Decision Making of Recurrent Prostate Cancer: A Prospective Crossover Trial
Source: Mol Imaging Biol. 2025 May 28;27(4):597–605. doi: 10.1007/s11307-025-02020-5 (PMC12405339; doi:10.1007/s11307-025-02020-5)
Supplement: Supplementary file 3 — Supplementary file3 (DOCX 20 KB) [file 11307_2025_2020_MOESM3_ESM.docx]

**Supplementary 3**

**Inclusion Criteria:**

- Biopsy-proven PCa,
- Patients that gave free written consent to participate in the study through the Informed Consent Form;
- Patients willing to comply with all study procedures and show availability for the duration of the study;
- Men with age between 18 and 80 years old at the time of enrolment;
- Patients diagnosed with prostate cancer that have followed a radical treatment by RPE and/or radiotherapy and/or systemic treatment consisting ADT and/or chemotherapy;
  - Biochemical relapse:
    - Patients with former RPE: the last two prostate specific antigen (PSA) measurements had values >0.2ng/mL;
    - PSA increase of 2.0 ng/ml above the nadir after radiotherapy.
  - Residual disease:
    - After RPE: positive PSA level after surgery;
    - After radiotherapy and/or systemic therapy: two consecutive PSA measurements >0.2 ng/ml (with attention to false-positive results).
- Patients that have a prescription to perform a [^18^F]-Fluorocholine PET/CT scan;
- Patients with PSA level <10 ng/mL at study inclusion;
- Patients with a life expectancy superior to 12 months;
- Patients with an Eastern Cooperative Oncology Group performance status scale (ECOG) ≤2;
- Patient under the Austrian National Healthcare System;
- Patients physically and psychologically able to participate in the study.

**Exclusion criteria:**

- Patients presenting known hypersensitivity to the active substance or any excipient of the investigational drug;
- Patients with alcohol or drug addiction, who have a serious illness, mental disorder or any other cause that could affect their participation in the study;
- unsuccessful randomization,
- PSA<0.2 at enrollment,
- Other known active malignancies.
