# Supplementary material for: [68Ga]Ga-PSMA-11 PET/CT and [18F]Fluorocholine PET/CT in Assessment and Clinical Decision Making of Recurrent Prostate Cancer: A Prospective Crossover Trial
Source: Mol Imaging Biol. 2025 May 28;27(4):597–605. doi: 10.1007/s11307-025-02020-5 (PMC12405339; doi:10.1007/s11307-025-02020-5)
Supplement: Supplementary file 4 — Supplementary file4 (DOCX 15 KB) [file 11307_2025_2020_MOESM4_ESM.docx]

**Supplementary 4:** Patients characteristics

| Parameter | Percentage (%) | Patients (n) |
| --- | --- | --- |
| Initial T stage | | |
| 1a | 0% | 0 |
| 2 (not specified) | 0% | 0 |
| 2a | 6.5% | 3 |
| 2b | 4.3% | 2 |
| 2c | 34.7% | 16 |
| 3 (not specified) | 0% | 0 |
| 3a | 34.7% | 16 |
| 3b | 17.4% | 8 |
| 4 | 0% | 0 |
| Tx | 2.2% | 1 |
| Initial N stage | | |
| 0 | 65.2% | 30 |
| 1 | 30.4% | 14 |
| Nx | 4.3% | 2 |
| Initial M stage | | |
| 0 | 91.3% | 42 |
| 1 | 8.7% | 4 |
| Mx | 0% | 0 |
| Gleason score | | |
| 6 | 4.3% | 2 |
| 7 (3+4) | 19.6% | 9 |
| 7 (4+3) | 23.9% | 11 |
| 8 | 28.3% | 13 |
| 9 | 15.2% | 7 |
| 10 | 4.3% | 2 |
| Not available | 4.3% | 2 |
| Initial Stage based on pathology of RPE | | |
| I (T1a N0 M0, or T2a N0 M0) | 2.2% | 1/46 |
| II (T2b N0 M0, or T2c N0 M0) | 28.3% | 13/46 |
| III (T3 N0 M0, or T4 N0 M0) | 34.7% | 16/46 |
| IV (Any T N1 M0, or any T any N M1) | 34.7% | 16/46 |
| Not available | 0% | 0 |
| tPSA | | |
| 0.5≥ tPSA | 28.3% | 13/46 |
| 0.5<tPSA≤1 | 30.4% | 14/46 |
| 1<tPSA≤2 | 10.9% | 5/46 |
| 2< tPSA | 30.4% | 14/46 |
| PSAdt | 76.1% | 35/46 |
| PSAdt≤6months | 42.8% | 15/35 |
| PSAdt>6months | 57.1% | 20/35 |
| Not available | 23.9% | 11/46 |
| PSAV (PSA Velocity) after RPE (ng/ml/year) | 78.3% | 36/46 |
| PSAV≤0.5 | 51.3% | 20/39 |
| 0.5<PSAV≤1 | 15.4% | 6/39 |
| 1<PSAV≤2 | 17.9% | 7/39 |
| PSAV>2 | 15.4% | 6/39 |
| Not available | 15.2% | 7/46 |
| Median PSA Velocity | 0.7 ng/ml/year | |
| Former Treatment | | |
| RPE | 100% | 46/46 |
| Radical Treatment (RPE±RT) | 58.7% | 27/46 |
| RPE±RT+ADT (ongoing) | 26.1% | 12/46 |
| RPE±RT+ADT (ongoing)+Chemotherapy | 15.2% | 7/46 |
| Recent PET/CT status | | |
| Both (PSMA & FCH) positive | 54.3% | 25/46 |
| Both (PSMA & FCH) negative | 21.7% | 10/46 |
| Only PSMA positive | 21.7% | 10/46 |
| Only FCH positive | 2.2% | 1/46 |
| Type of recurrence | | |
| All recurrences based on recent PET/CT | 78% | 36/46 |
| Only local recurrence based on recent PET/CT | 4.3% | 2/46 |
| Regional recurrence based on recent PET/CT | 15.2% | 7/46 |
| Metastatic recurrence based on recent PET/CT | 59% | 27/46 |
