# Supplementary material for: [68Ga]Ga-PSMA-11 PET/CT and [18F]Fluorocholine PET/CT in Assessment and Clinical Decision Making of Recurrent Prostate Cancer: A Prospective Crossover Trial
Source: Mol Imaging Biol. 2025 May 28;27(4):597–605. doi: 10.1007/s11307-025-02020-5 (PMC12405339; doi:10.1007/s11307-025-02020-5)
Supplement: Supplementary file 5 — Supplementary file5 (DOCX 20 KB) [file 11307_2025_2020_MOESM5_ESM.docx]

**Supplementary 5:** Paraclinical and PET/CT findings in metastatic patients

| Metastasis extent | N | low-volume based on CHAARTED criteria* | Tx change** | tPSA<1 | 1<tPSA<2 | tPSA>2 | PSMA & FCH PET/CT +ve | Only PSMA PET/CT +ve | Only FCH PET/CT+ | PSAdt≤6m | PSAdt>6m | PSA Velocity>0.5  After RPE |
| --- | --- | --- | --- | --- | --- | --- | --- | --- | --- | --- | --- | --- |
| Oligometastatic (≤3 lesions) | 14 | 12 | 1 | 7 | 2 | 5 | 9 | 5 | 0 | 5 (4 of them with tPSA<1) | 6 | 12 |
| Polymetastatic (4-10 lesions) | 13 | 7 | 1 | 5 | 3 | 5 | 11 | 2 | 0 | 7 (3 of them with tPSA<1) | 3 | 3 |
| Metastatic (total) | 27 | 19 | 2 | 12 | 5 | 10 | 20 | 7 | 0 | 12 | 9 | 15 |

*(+ve = positive)*

* CHAARTED criteria (high-volume disease:≥4 bone metastases, at least one of them outside vertebral or pelvic bones and/or visceal metastasis)

** Treatment change from radical alone to systemic therapy (starting ADT)
